# Has-miR-300—GADD45B promotes melanoma growth via cell cycle

**DOI:** 10.18632/aging.205276

**Published:** 2023-12-07

**Authors:** Long Chen, Chenglong Fang, Xiaoxue Yuan, Mengqi Liu, Ping Wu, Li Zhong, Zhiyong Chen

**Affiliations:** 1 Department of Burn Plastic and Cosmetology, Affiliated Fuling Hospital, Chongqing University, Chongqing 408099, China; 2 College of Bioengineering, Chongqing University, Chongqing 400000, China; 3 Department of Immunology, School of Basic Medical Sciences, Chengdu Medical College, Chengdu 610500, Sichuan, China; 4 Non-Coding RNA and Drug Discovery Key Laboratory of Sichuan Province, Chengdu Medical College, Chengdu 610500, Sichuan, China; 5 Department of Rehabilitation, LinYi People’s Hospital, Linyi 276000, Shandong, China

**Keywords:** miR-300, GADD45B, CDKN1A, cell cycle, melanoma

## Abstract

Response to oncogenic factors like UV, GADD45 family in skin participates in scavenging ROS, DNA repair and cell cycle control. Because of this, the previous study of the chronic UVB injury model has found that hsa-miR-300 can conduct intercellular transport by exosomes and target regulation of GADD45B. Whether the hsa-miR-300—GADD45B still regulates tumor development by cell cycle pathway is unclear. Through transcriptomic analysis of primary (n=39) and metastatic (n=102) melanoma, it was confirmed that in metastatic samples, some of the 97 down-regulated genes participate in maintaining skin homeostasis while 42 up-regulated genes were enriched in cancer-related functions. Furthermore, CDKN1A, CDKN2A, CXCR4 and RAD51 in the melanoma pathway, were also differentially expressed between normal skin and melanoma. CDKN1A and CDKN2A were also found to be involved in TP53-dependent cell cycle regulation. In conclusion, it was speculated that CDKN1A, CDKN2A, TP53, GADD45B and hsa-miR-300 may have regulatory relationships. It was demonstrated that there is a bidirectional regulation between hsa-miR-300 and TP53. In addition, miR-300 can regulate CDKN1A by GADD45B/TP53 and promote melanoma growth by accelerating the cell cycle transition from G1/S to G2 phase.

## INTRODUCTION

Studies have confirmed that miRNA expression disorders occur in a variety of cancer types [1–3]. A large number of differentially expressed miRNAs were also detected in melanoma, which may be related to the abnormal expression of key miRNA maturation proteins AGO, TARBP2 and SND1. At present, most studies on miRNA in melanoma are aimed at screening differential expression profiles as specific marker molecules, but the specific biological effects and related mechanisms of miRNA remain to be further explored [4, 5]. These abnormal miRNAs can promote the growth of melanoma and may play a role in the drug resistance of melanoma cells [6, 7]. Compared with normal melanocytes, some miRNA expressions are inhibited in melanoma, suggesting that miRNA may act as a tumor suppressor. Let-7 family is generally down-regulated in melanoma, and let-7b, a member of this family, has been reported to directly target cyclin D1 controlling cell cycle progression by activating cyclin-dependent kinases 4 and 6 (CDK4/6) [8]. However, some miRNAs promote tumor growth and show a downregulation trend in melanoma due to special regulatory pathways. In this study, miR-300 was down-regulated in melanoma, and whether miR-300 is further involved in promoting melanoma growth and malignant transformation remains to be further explored.

The GADD45 family also plays a role in regulating tumor development. Gadd45a^-/-^ and Gadd45b^-/-^ mice show increased mutation frequency and susceptibility to ionizing radiation and chemical carcinogens [9]. In addition, inhibition of GADD45A and GADD45G expression is critical to the survival of cancer cells [10] the mutation frequency and specific functions of GADD45 family members in different types of cancer remain to be determined. Because of the GADD45 family in stress reaction and the role of the carcinogenic process, the family protein is likely to be ultraviolet-induced and also plays an important role in the pathogenesis of melanoma. Studies have shown that after a certain dose of UVB irradiation, only GADD45A significantly upregulated among many known downstream genes of TP53 only in melanocytes while there is no change in GADD45 expression in fibroblasts and keratinocytes [11]. Similarly, some studies have also found that GADD45 family proteins play an important role in human immunity. In addition to regulating the differentiation and growth of immune cells, it also affects the expression and secretion of immune effector factors [12, 13]. In melanoma, whether GADD45B can regulate tumor growth through cell proliferation, cycle apoptosis migration and other functions needs further confirmation.

## MATERIALS AND METHODS

### Cell culture and animal model

Melanoma line (A375) and 293T cell line were obtained from ATCC. Cells were maintained in DMEM supplemented with 10% fetal bovine serum (FBS, Gibco), 100 U/ml penicillin and 100 mg/ml streptomycin (Invitrogen, USA), at 37° C and 5% CO2. The cell lines were free of mycoplasma contamination and were authenticated by short tandem repeat analysis as described [14]. The shRNAs against miR-300, TP53, GADD45B, CDKN1A and CDKN2A were listed in Table 1. Generation of viral particles and stable transduction were performed as described [15].

**Table 1:** Primers used for expression vector and interference vector construction.

**Gene**	**Primer sequences**
TP53-shRNA-1^st^	5'- GATCCCCCGGCGCACAGAGGAAGAGAATTTCAAGAGA -3'
TP53-shRNA-2^nd^	5'- ATTCTCTTCCTCTGTGCGCCGTTTTTA -3'
TP53-shRNA-3^rd^	5'- ATTCTCTTCCTCTGTGCGCCGGGG -3'
TP53-shRNA-4^th^	5'- AGCTTAAAAACGGCGCACAGAGGAAGAGAATTCTCTTGAA-3'
pCDNA- TP53-F	5'- GGGGTACCCCTCTGACTGCGGCTCCTCCAT -3'
pCDNA- TP53-R	5'-CTTCCCGGACTGAGTCTGACTCGGAATTCCG-3'
CDKN1A-shRNA-1^st^	5'- GATCCCCGACAGATTTCTACCACTCCAATTCAAGAGA -3'
CDKN1A-shRNA-2^nd^	5'- TTGGAGTGGTAGAAATCTGTCTTTTTA -3'
CDKN1A-shRNA-3^rd^	5'- TTGGAGTGGTAGAAATCTGTCGGG -3'
CDKN1A-shRNA-4^th^	5'- AGCTTAAAAAGACAGATTTCTACCACTCCAATCTCTTGAA -3'
pCDNA-CDKN1A-F	5'- GGGGTACCCCAGCCGGTTCTGACAT -3'
pCDNA-CDKN1A-R	5'- TTAGGGCTTCCTCTTCGGAATTCCG-3'
CDKN2A-shRNA-1^st^	5'- GATCCCCGCTCTGAGAAACCTCGGGAAATTCAAGAGA -3'
CDKN2A-shRNA-2^nd^	5'- TTTCCCGAGGTTTCTCAGAGCTTTTTA -3'
CDKN2A-shRNA-3^rd^	5'- TTTCCCGAGGTTTCTCAGAGCGGG -3'
CDKN2A-shRNA-4^th^	5'- AGCTTAAAAAGCTCTGAGAAACCTCGGGAAATCTCTTGAA -3'
pCDNA-CDKN2A-F	5'- GGGGTACCCCCGCCGCCGGCTCCAT -3'
pCDNA-CDKN2A-R	5'-ACAATCGGGGATGTCCGGAATTCCG-3'

All animals used were pathogen-free Balb/c severe combined immunodeficient (SCID) mice aged 9-14 weeks with a weight of 25-30 g in this study. The mice were provided food and water ad libitum and their condition was monitored daily. Apart from visible tumors, the general condition of the animals was evaluated by movement/behavior, weight development, food and water intake and fur condition. Finally, the mice were killed by cervical dislocation after having been anesthetized. The experiment was carried out as previously described [16]. Briefly, 5 scid mice in each group received subcutaneous injections of 10^6^ melanoma cells (A375) with miR-300/CDKN1A knockdown and overexpression, respectively. Mice with ulcerated tumors or tumors with an estimated mass exceeding 10% of the animal’s weight were euthanized immediately. 1 and 2 animals were lost to murine lymphoma in the miR-300(+)CDKN1A(+) and miR-300(-)CDKN1A(-)group. Taken together, 3 animals from each group were included in the further analyses. The methodology for carrying out the animal experiments was consistent with the guidelines for the welfare of animals in experimental neoplasia. The experiment was recommended and supervised by the institutional animal welfare officer and approved by the local licensing authority (The Ethics Committee of Chongqing University Three Gorges Hospital).

### Bioinformatics analysis of melanoma samples

Melanoma samples used in this section are based on data from two databases, respectively is GEO and TCGA database. The TCGA melanoma data set in the database have been described in detail in the section in the last chapter, there is no need to do. And in this chapter, the experiment also used a clinical melanoma sample derived from the GEO database sequencing data (GSE59455). 196 samples in this dataset were collected during clinical diagnosis by pathology Services of New South Wales, Australia between 2004 and 2009. The samples were immobilized with formalin and paraffin-embedded. The study was approved by the local ethical Society For stage II or more malignant tumors, including both *in-situ* and metastatic melanomas [17]. Due to the surgical isolation of tumor samples and their subsequent preservation, as well as the lack of purity resulting from mishandled RNA extraction in different batches. In addition, the need to access sufficient tumor tissue to allow matching genomic sequencing, gene expression and immunohistochemical analysis required that cases with a thin primary melanoma (Breslow thickness less than 2mm) with only a small volume of metastatic disease (locoregional or distant metastases) were excluded from the study. RNA was successfully extracted from 157 (80%) tumor samples and clinical information is summarized in Table 2.

**Table 2:** Clinical parameters.

**Clinical parameter**	**Total patient no. (%)**
Total	157 (100)
**Malignancy**	
Primary	39 (24.8)
Metastatic	102 (65.0)
Unknown	16 (9.2)
**Sex**	
Female	48 (30.6)
Male	109 (69.4)
**Gene subtype**	
BRAF	35 (22.3)
NRAS	33 (21.0)
WT	73 (46.5)
Unknown	16 (9.2)
**Age at 1st Diagnosis**	
Mean (range)	65.8 (23.3 - 94.5)
Unknown	15 (9.6)
**Survival (weeks)**	
Mean (range)	206.6 (3.1 - 1418)
Alive	17 (10.8)
**Breslow Thickness**	
Mean (range)	5.3 (0.4 - 33)
No. Unknown	62 (39.5)
**Solar Elastosis**	
None	8 (5.1)
Mild	25 (15.9)
Moderate	17 (10.8)
Severe	42 (26.8)
Unknown	65 (41.4)
**Clark’s Level (Stage)**	
1	16 (10.2)
2	47 (29.9)
3	47 (29.9)
4	21 (13.4)
**Weeks Local to Distal Metastasis**	
Mean (range)	121 (5.6 - 343.4)
No. Unknown	66 (42)

### Microarray data and enrichment analysis

Total RNA from tissues, cells and exosomes was isolated using Trizol extractions (Invitrogen, USA). The RNA quantity was assessed by NanoDrop®ND-1000 spectrophotometer and RNA 6000 NanoChips with the Agilent 2100 Bioanalyzer (Agilent, Palo Alto, CA, USA). On the one hand, 100 ng of total RNA was amplified using the Ambion® WT Expression Kit (4411973, Life Technologies, USA). 5.5 μg of the cDNA was fragmented and labeled with the GeneChip® WT Terminal Labeling kit (901525, Affymetrix, USA). On the other hand, small RNA libraries were prepared using 1μg of total RNA according to the TruSeq Small RNA Sample Preparation Guide (Illumina, San Diego, CA, USA). Libraries were sequenced either on Illumina HiSeq 2000 or HiSeq 2500 using v3 chemistry. To generate count data, the raw sequences were compared to mouse mature miRNA sequences (from miRBase version 17) and non-coding RNA sequences (Rfam version 10) by MEGABLAST.

Background deletion, quantile normalization, and probe assembly were performed. Differently expressed genes (DEGs) between control vs. treatment samples were detected by the empirical Bayes method [18, 19] while DEMs were detected by the R package DESeq [20]. P-values were adjusted for multiple comparisons using the Benjamini-Hochberg procedure [21]. Genes with an adjusted p-value of ≤ 0.01 and | logFC |≥ 1.0 were considered as differentially expressed. Gene enrichment analyses were performed with DAVID version 6.7 (https://david.ncifcrf.gov/). The enriched biological GO and pathway terms were identified [22]. The interaction network was drawn by Cytoscape. Some other databases used are listed in Table 3.

**Table 3:** List of databases.

**Database ID**	**URL**
GEO Dataset	https://www.ncbi.nlm.nih.gov/gds/?term=
TCGA	https://www.cancer.gov/
cBioportal of cancer genomics	https://www.cbioportal.org/
FireBrowe	http://firebrowse.org
DSA	http://cancer.digitalslidearchive.net/
The Human Protein Atlas	https://www.proteinatlas.org/
Expasy	http://web.expasy.org/protparam/
PSIPRED	http://bioinf.cs.ucl.ac.uk/psipred/
Mexpress	https://mexpress.be/
Ensemble	http://asia.ensembl.org/index.html
Linked Omics	http://www.linkedomics.org/
TransmiR v2.0	http://www.cuilab.cn/transmir
Targetscan	http://www.targetscan.org/vert_72/
OncomiR	http://www.oncomir.org/oncomir/index.html
TIMER	https://cistrome.shinyapps.io/timer/
STRING	https://string-db.org/
GEPIA	http://gepia.cancer-pku.cn/index.html
Pathview	https://pathview.uncc.edu/
TIP	http://biocc.hrbmu.edu.cn/TIP/index.jsp

### RT-qPCR

Total RNA was extracted using TRIzol® reagent (Takara Bio, Inc., Japan), and RNA concentration and purity were determined. Total RNA was reverse transcribed into cDNA (10μg) using the PrimeScript™ RT reagent kit (Takara Bio, Inc.), according to the manufacturer’s protocol. The reaction conditions of the reverse transcription of RNA into cDNA were: Incubation at 25° C for 10 min, additional incubation at 42° C for 30 min, and heating at 95° C for 5 min. cDNA was diluted with DEPC water. Fluorescent RT-qPCR was performed following the manufacturer’s protocol (Thermo Fisher Scientific, Inc., USA). The primers were designed and synthesized by Chongqing Life Biological Technology, Ltd. The reaction system volume was 25 μl and consisted of cDNA (1 μl), 10X PCR buffer (2.5 μl), 10 mmol/l dNTPs (2 μl), PCR upstream primers (1 μl), PCR downstream primers (1 μl), Taq DNA polymerase (1 μl), and deionized water (16.5 μl). The reaction conditions were as follows: Pre-denaturation at 95° C for 5 min, denaturation at 94° C for 1 min, annealing at 54° C for 45 sec, extension at 72° C for 1 min, in a total of 30 cycles, followed by extension at 72° C for 10 min. β-actin was used as the internal reference. The experiment was repeated 3 times independently.

### Western blotting

Proteins were extracted from cells and quantified using BCA protein assay kit (Thermo Scientific-Pierce, Rockford, IL, USA). Equal amounts (20–25 μg) of protein were separated by 10% SDS-PAGE polyacrylamide gels (Bio-Rad, USA) and then transferred to PVDF membranes (Millipore, Nottingham, UK). After blocking in a solution of 5% nonfat dry milk diluted in Tris-buffered saline for 1 h, the blots were incubated with primary antibodies and then with horseradish peroxidase-conjugated secondary antibodies. Bound antibodies were detected using the enhanced chemiluminescence (Pierce, USA) and the ECL Western blotting detection system (Millipore, USA).

### Cell cycle analysis

Flow cytometry takes advantage of the ability of the fluorescent DNA probe propyl iodide (PI) to embed in the base pairs of double-stranded DNA, and indirectly measures the amount of DNA in a single cell by using a machine to detect the intensity of fluorescence emitted by PI. Digested the treated cells with pancreatic enzymes, collected the cells by centrifugation at low speed for 5 min, and removed the supernatant. The PBS buffer was pre-cooled on ice for 20 min in advance, and then the PBS was slowly added along the wall of the centrifuge tube, gently turned the centrifuge tube, and the cell precipitation was washed three times. Cell precipitation was then suspended with about 300 μL PBS buffer, and 700 μL pre-cooled anhydrous ethanol was added, mixed upside down, and the cells were fixed at -20° C for more than 24 h. The cells were washed and precipitated with PBS 3 times. Add 100 μL RNaseA(1mg/mL) into the cells, incubate at 37° C for 30 min, and then add 400 μL PI solution (50 mg/L) to make the final concentration of PI in the cell solution 40 mg/L. After standing for 10 min away from light, the samples were detected by flow cytometry. DNA content analyses were conducted using a FACS Canto II flow cytometer (BD Biosciences) and analyzed using FlowJo software.

### Tumor subtype classification

Raw counts and corresponding clinical information of 470 melanoma transcriptome sequencing data were obtained from the TCGA dataset. Then, the R software packages ConsensusClusterPlus (V1.54.0) was using for consistency analysis [23] with the maximum number of clustering as 6. Repeat 100 times to extract 80% of the total samples, clusterAlg = “hc” and innerLinkage=‘ward.D2’. Briefly, we prepared the input data at first. Rows represent genes and columns represent sample expression data. In order to obtain the best clustering effect, genes can be screened. The matrix was normalized. Then, run ConsensusClusterPlus and select 80% of the samples and genes for repeated sampling. Finally, collect cluster-consensus and item-consensus matrices. The clustering heat maps were analyzed by R software package pheatmap (v1.0.12) [24]. Gene expression with variance above 0.1 were reserved in the heatmap. When more than 1000 target genes were input, the genes were sorted in descending order and the top 25% were selected for survival and prognostic analyses of each subtype. Visualization analysis was performed by R software packages Survival and SurvMiner [25]. All the above analysis methods were implemented by R V4.0.3.

### Statistical analyses

Results are presented as mean values ± standard error of the mean (SEM). Unless mentioned otherwise, the statistical comparison between groups was performed by using t-test, a maximum of three comparisons were performed per panel, and robustness of statistical significance was verified after correction for multiple testing. Probability was considered to be significant at p-value < 0.05.

### Availability of data and materials

The supporting data can be acquired via correspondence author.

### Consent for publication

All the authors were consented to publication.

## RESULTS

### Molecular differences between *in-situ* and metastatic melanoma

Tumor development is a dynamic continuous biological process, carcinogenic factors can produce different tumor cells with different genotypes and directly affect the subsequent tumor malignant degree and treatment difficulty [26, 27]. In the clinical diagnosis, often according to tumor location and the malignant degree of cancer cells could be divided into primary melanoma and metastasis melanoma [28, 29]. There is a large difference in these two types of melanoma formation reasons.

**Figure 1:**
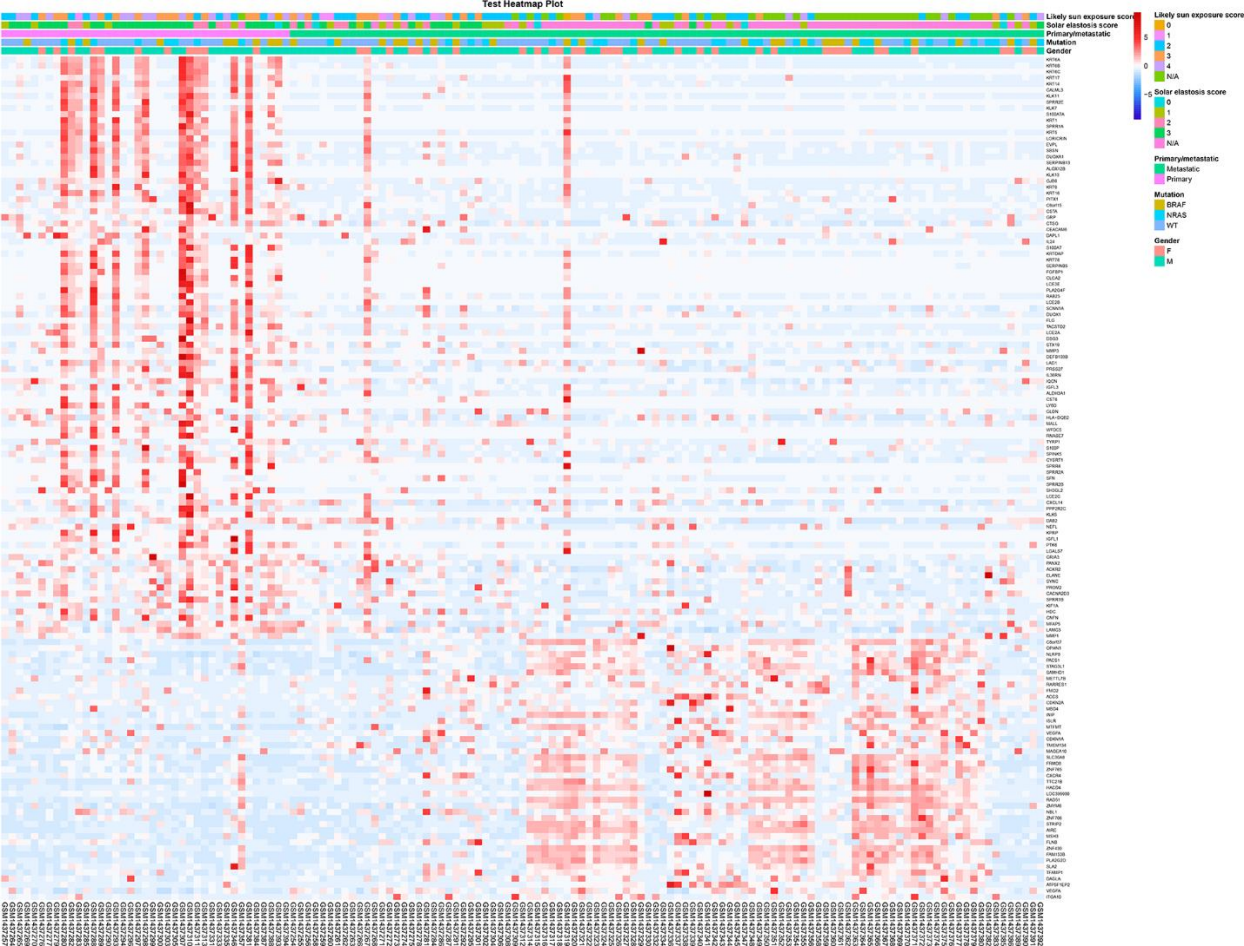
**The screening and functional analysis of DEGs among primary and metastatic melanomas.** The different expression genes between patients with primary and metastatic melanoma (heat map). The red points and blue points represented high and low expression level, respectively. The clinical characters of all samples were shown on the top (likely sun exposure score, solar elastosis score, primary/metastatic, mutation and gender).

The transcriptome sequencing analysis used data from the GEO database. In the previous study, the data quality for the RNA was checked using principal component analysis (PCA) and outlying melanomas were removed before further analyses, resulting in 141 melanomas. Among them, including 39 primary and 102 metastatic malignant melanoma samples. | logFC |> 1.0 and p-value <0.05 as screening conditions, it was found that compared with *in-situ* melanoma, there were 97 significantly down-regulated genes and 42 significantly up-regulated genes in metastatic melanoma (Figure 1 and Supplementary Figures 1, 2). It was found that up-regulated genes were enriched in multiple cancer-related functions, while down-regulated genes are involved in maintaining skin homeostasis. In metastatic melanoma, the body may up-regulate cancer-related genes to increase the degree of malignancy of tumor cells [30, 31]. At the same time, by down-regulating, the gene set that maintains the homeostasis of skin tissue [32, 33], the strength of skin tissue structure can be changed so that the malignant tumor cells can easily metastasize to other sites.

### Key genes and clinical value in melanoma pathways

**Figure 2:**
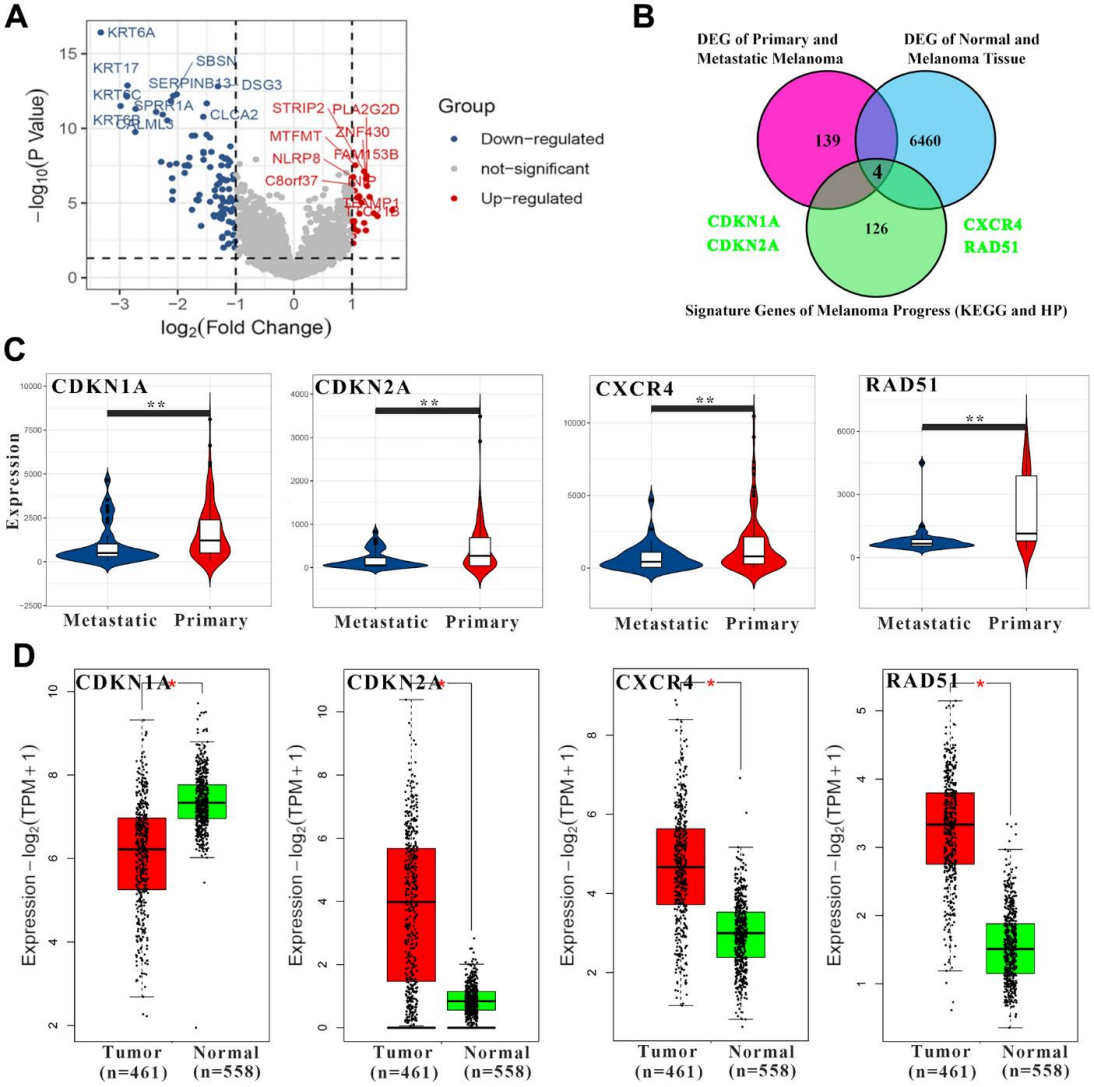
**The screening of key genes in the melanoma pathway.** (**A**) The volcano map shows differential genes between primary and metastatic melanoma. The red points represent up-regulated genes and the blue ones represent down-regulated genes in the metastatic samples. The top 10 up- and down-regulated genes with the highest significance were labeled. (**B**) The Venn diagram shows screening conditions for key genes. (**C**) The expression levels of 4 key genes in melanoma. (**D**) The expression levels of 4 key genes in normal and melanoma tissues. * p-value < 0.05, ** p-value < 0.01, *** p-value < 0.001.

Molecular differences in different types of melanoma may exist in the tumorigenesis stage, not acquired in the development stage. To prove this point, by analyzing the TCGA database in the melanoma and transcriptome data of normal skin tissues, 6464 DEGs were screened. In addition, a total of 143 DEGs were screened in comparison between primary and metastatic melanoma (Figure 2A). There were 130 correlated genes in melanoma pathway (according to KEGG and HP databases) (Figure 2B). Among them, CDKN1A, CDKN2A, CXCR4 and RAD51 were significantly down-regulated in metastatic melanoma when compared with the primary type (Figure 2C). Meanwhile, CDKN2A, CXCR4 and RAD51 were significantly up-regulated in melanoma compared with normal skin except CDKN1A (significantly down-regulated) (Figure 2D). Therefore, it is speculated that these four genes may play an important role in the occurrence and development of melanoma. Notably, both CDKN1A and CDKN2A belong to the same family (cyclin dependent kinase inhibitor) and show the same change trend in the tumor development stage while with different trends in the development stage. The main reasons for this phenomenon may be due to the different positions of CDKN1A and CDKN2A in the melanoma pathway and cell cycle pathway, as well as differences in upstream regulatory networks (eg. TP53 and GADD45B). To fully understand the role of CDKN1A, CDKN2A, CXCR4 and RAD51 in clinical melanoma patients, a genetic panorama with relevant clinical information (mutation spectrum, sex, diagnosis age, American Joint Committee on Cancer Metastasis Stage Code, overall survival, disease free, American Joint Committee on Cancer Tumor Stage Code) was drawn. In melanoma patients, CDKN1A (14%) and CDKN2A (34%) had a very high frequency of genetic aberration (inframe mutation, missense mutation, truncating mutation, amplification, deep deletion, mRNA high, mRNA low, protein high and low). In addition, it was also found that CDKN1A and CXCR4 have high levels of methylation (Supplementary Figure 3A). Both methylation and mutation may change expression levels. It may also explain why CDKN1A and CDKN2A, which belong to the same family, show different expression trends. Furthermore, the receiver operating characteristic (ROC) curves and survival curves were calculated (Supplementary Figure 3B, 3C). It was shown that only RAD51 had good clinical predictive properties (area under curve value=79.0096) while only CXCR4 (lower level, p-value=0.021) and RAD51 (higher level, p-value=0.027) could lead to poorer prognosis.

### CDKN1A and CDKN2A are involved in cell cycle pathway of melanoma

In cancer research, 14 classical signaling pathways directly regulate several major functions of cancer cells, including apoptosis, cell cycle, migration and DNA repair. These functions are often used to evaluate the degree of malignancy of tumor cells and the key genes.

**Figure 3:**
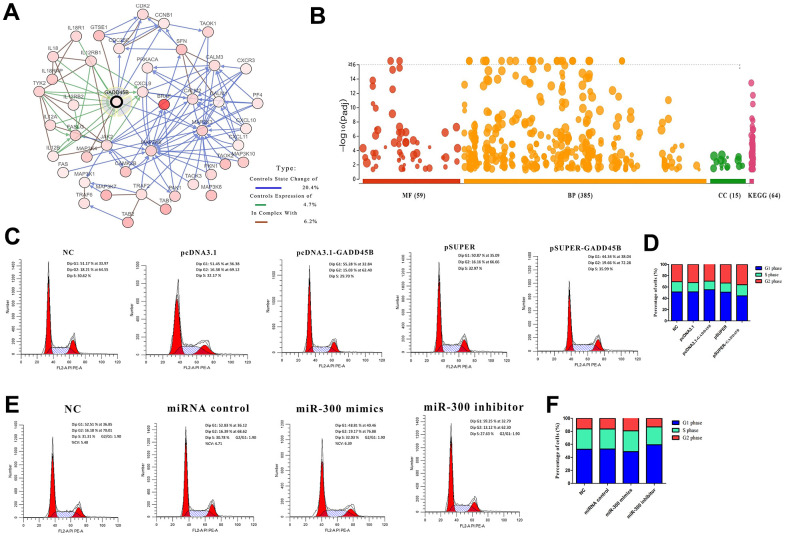
**GADD45B regulated cell cycle in melanoma.** (**A**) The associated molecular network of GADD45B in melanoma. Different colored arrows represent different types of action. The legend is listed on the right. (**B**) The enrichment analysis of GADD45B associated network. (**C**–**F**) Flow cytometry was used to detect the proportion of A375 in each stage of cell cycle. Different absorption peaks represent different stages of cell cycle, and the area under each absorption peak curve represents the proportion of cells in this period. The proportion of cells in each period was listed on the right side of the peak diagram after statistics, showing the proportion of cells in G1, S and G2 phases respectively. At the same time, the proportion of cells in each group at different stages was statistically analyzed by corresponding histograms. Blue represents G1 phase, green represents S phase, and red represents G2 phase. * p-value < 0.05, ** p-value < 0.01, *** p-value < 0.001.

In our previous study on melanoma [34], GADD45B, a key protein regulating melanoma development was screened with miR-300, an upstream regulatory element. A molecular interaction network centered on GADD45B was constructed with 44 genes based on the cBioPortal and String database (Figure 3A). By constructing a melanoma prognosis model based on the expression profiles of molecular network genes, the patient cohort can be successfully divided into high risk (median time=4.1) and low risk group (median time=11.5). It might be suggested that GADD45B was a significant factor which regulate prognosis in melanoma (LogRank p-value=4.82e-10, HR=2.395, 95%CI (1.819, 3.153)) (Supplementary Figure 4). To explore the specific biological effects of this molecular network in melanoma, enrichment analyses were performed using the DAVID Database and found that these genes may be involved in a total of 385 BP, 15 CC, 59 MF and 64 KEGG pathways, such as cell proliferation, migration, apoptosis and cell cycle (Figure 3B). Transfected GADD45B expression or interference vectors and miR-300 mimics or inhibitors into A375 cells, the proportion of cells in G1, S, and G2 phases were detected via flow cytometry. Statistically, when transfected with GADD45B expression vector or miR-300 inhibitors to up-regulate intracellular GADD45B level, the number of cells in G1 phase significantly increased (55.28% and 59.25%) (p-value<0.05), and the number of cells decreased in S phase (29.70% and 27.63%) and G2 phase (15.03% and 13.12%) (p-value<0.05). Conversely, when transfection of GADD45B interfering vector or miR-300 mimics decreased the level of GADD45B in melanoma cells, the number of cells in G1 phase decreased (44.34% and 48.81%) (p-value<0.05), and the cell volume increased in S phase (35.99% and 32.03%) and G2 phase (19.66% and 19.17%) (p-value<0.05), indicating that the proportion of cells undergoing DNA replication and mitosis increased (Figure 3C–3F). Therefore, it was suggested that miR-300 may promote cell cycle progression in melanoma via GADD45B.

**Figure 4:**
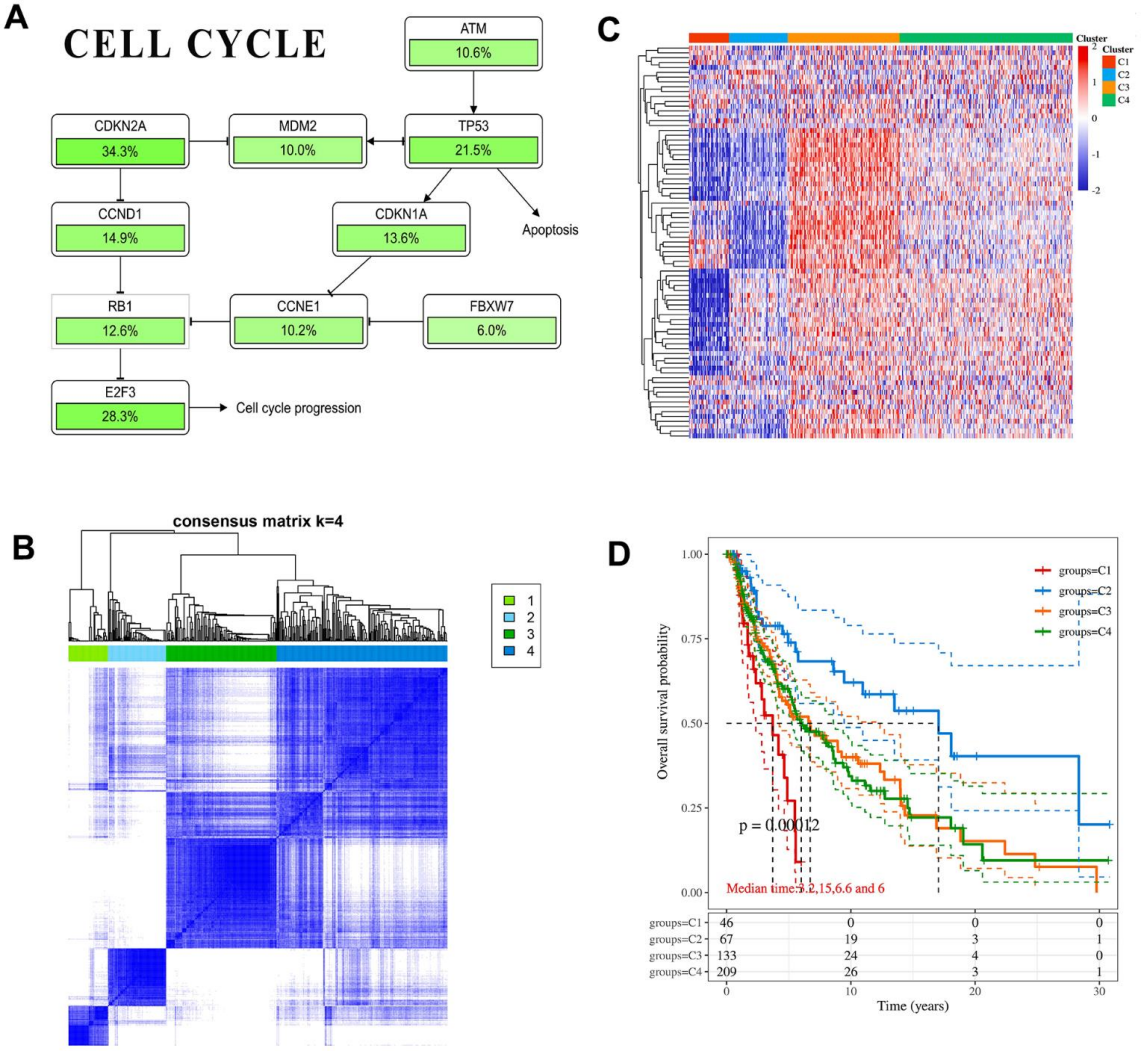
**CDKN1A and CDKN2A participate in cancer correlation pathways.** (**A**) The schematic diagram of pathways includes key gene mutual regulation. The mutation ratio of each gene in the melanoma sample was marked below. (**B**) Heatmap depicting consensus clustering solution. (**C**) The heatmap of cell cycle-related gene expression in different subgroups, red represents high expression and blue represents low expression. (**D**) Kaplan-Meier survival analysis of the different subtypes.

Through literature review and comparison of core genes in pathways, only CDKN1A and CDKN2A are involved in cell cycle pathways (Figure 4A and Supplementary Figure 5A, 5B) [35, 36]. In addition, CDKN1A is regulated by TP53, and CDKN2A can inhibit TP53 expression through MDM2. Immunohistochemical analysis showed that compared with normal tissues, the protein level of CDKN1A was significantly down-regulated in melanoma, while the CDKN2A protein level was significantly up-regulated (Supplementary Figure 5C). It was consistent with the trend of mRNA level. However, CDKN1A and CDKN2A expression differences have not been found in different subtypes of melanoma (Supplementary Figure 5D), suggesting that CDKN1A and CDKN2A may become new type markers. The melanoma patients in TCGA were divided into 4 subtypes by cell cycle pathway-related genes (Figure 4B). After cluster analysis, significant differences in molecular expression were identified (Figure 4C). The median survival time of each subtype in the 4 groups was 3.2 years, 15 years, 6.6 years and 6 years, respectively (Figure 4D). The prognosis of subtype 1 was the worst, and the prognosis of subtype 2 was significantly better than that of other patients (p-value =0.00012). It indicated that in patients with melanoma when cell cycle pathways are activated to different degrees, the expression changes of pathway-related genes are different, which will directly affect the development of melanoma.

### The relationship among CDKN1A, CDKN2A, TP53 and GADD45B

**Figure 5:**
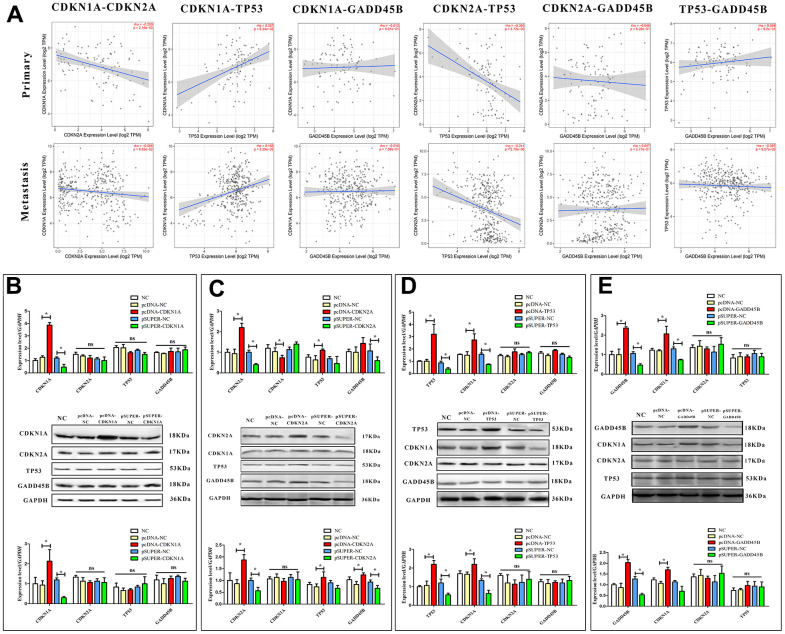
**Regulatory relationships among CDKN1A, CDKN2A, TP53 and GADD45B.** (**A**) The correlation between CDKN1A, CDKN2A, TP53, and GADD45B in primary and metastatic patients, respectively. (**B**–**E**) The expression or interference vector was used to alter the expression of CDKN1A, CDKN2A, TP53 and GADD45B in A375 respectively, and the expression of other genes was detected from the mRNA and protein levels. * p-value < 0.05, ** p-value < 0.01, *** p-value < 0.001.

As a key regulator of the cell cycle, TP53 has been shown to be involved in regulating the expression of GADD45 family proteins by CATG motifs in cell-cycle arrest and DNA repair [37]. Obviously, TP53 and CDKN1A/CDKN2A are in the same signaling pathway, and there is a definite regulatory relationship [35, 38]. Thus, it was supposed that CDKN1A, CDKN2A, TP53 and GADD45B may be involved in the regulation of the melanoma cell cycle, but the specific regulatory relationship between each factor needs to be further confirmed. To this end, we first conducted correlation statistics on primary and metastatic melanoma according to the expression levels of each gene in clinical samples. It was found that TP53 was negatively correlated with CDKN2A in both types of melanoma, while it was positively correlated with CDKN1A. A negative correlation between CDKN1A and CDKN2A was found, especially in primary melanoma. (Figure 5A) To further confirm the accuracy of correlation analysis and clarify the regulatory relationship, expression and interference vectors of each gene were transfected into melanoma cells respectively. The expression changes of other genes were detected from the mRNA and protein levels. Finally, CDKN2A may be located upstream of the signal axis, positively regulating the expression of TP53 and GADD45B. CDKN1A is located downstream of TP53 and GADD45B. Interestingly, CDKN1A was found to be negatively regulated by CDKN2A on mRNA level in A375 cells, but there was no significant difference in protein level. It was also confirmed that TP53 and GADD45B may not have a regulatory relationship (Figure 5B–5E).

### TP53 and miR-300 are mutually regulated

**Figure 6:**
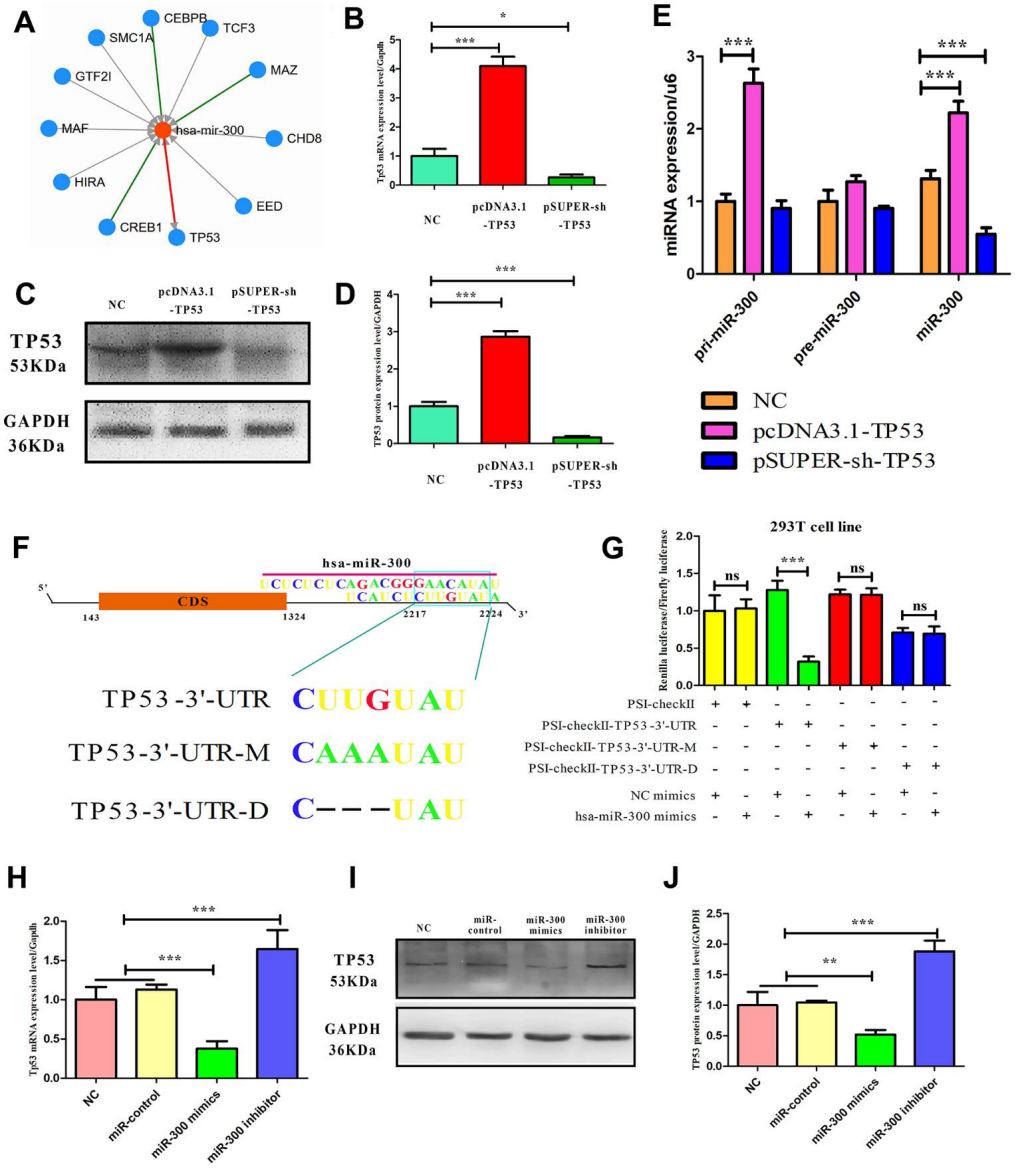
**The mutual regulatory relationship between hsa-miR-300 and TP53.** (**A**) A transcription factor-gene-miRNA network centered on hsa-miR-300. Arrows indicate regulatory relationships. (**B**–**D**) The ability of expression and interference vectors to regulate TP53 expression in A375 cells from mRNA and protein levels, respectively. (**E**) In A375 cells, TP53 vector affected the expression of hsa-miR-300. (**F**) Schematic diagram of miR-300 regulating site in TP53 UTR. “UTR-M” represents the mutation site and “UTR-D” represents the deletion site. (**G**) In 293T cell line, dual-luciferase reporting assay confirmed that hsa-miR-300 regulates TP53. (**H**–**J**) In A375 cells, hsa-miR-300 regulates the expression level of TP53. * p-value < 0.05, ** p-value < 0.01, *** p-value < 0.001.

It has been confirmed that CDKN1A and CDKN2A are involved in TP53-dependent cell cycle regulation, and CDKN1A and CDKN2A are located in the downstream and upstream of TP53, respectively. It is well known that TP53 plays a role in cancer mainly because of its transcriptional regulatory activity. The interaction of transcription factors, miRNA and target genes in melanoma was predicted by the TargetScan database and JASPAR database, and it was found that TP53 and miR-300 may have a mutual regulatory relationship (Figure 6A). Transfected with TP53 expression vector and interference vector, it was found that TP53 mRNA and protein levels were up-regulated and down-regulated in melanoma cells, respectively (Figure 6B–6D). Transfection of the TP53 expression vector and interference vector into melanoma cells, respectively, significant upregulation of miR-300 mature body was detected (1.4-fold, p-value<0.05) or down-regulated (0.4 fold, p-value< 0.05) (Figure 6E). In addition, targeting sequence prediction and dual-luciferase reporting assay confirmed the existence of miR-300 binding sites in the 3’-UTR region (2217-2224bp) of TP53 (Figure 6F, 6G). When this locus of action is mutated or deleted using genetic engineering techniques, the change of the double luciferin ratio is not detected when the intracellular miR-300 levels are changed again. Transfection of miR-300 mimics or reverse sequences can also regulate the protein expression level of TP53 in melanoma cells (p-value< 0.05) (Figure 6H–6J).

### MiR-300 down-regulate CDKN1A

**Figure 7:**
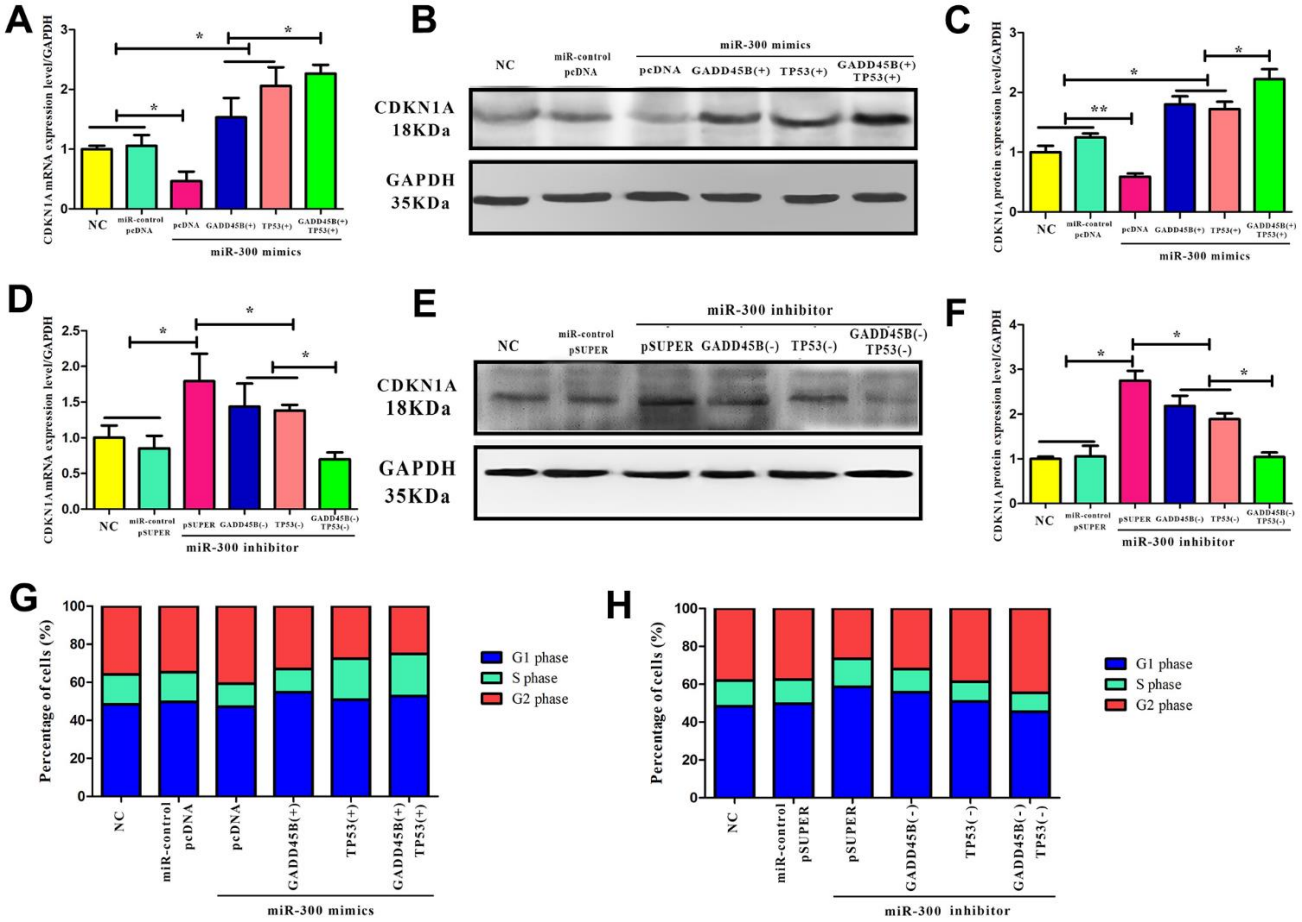
**CDKN1A was regulated by hsa-miR-300 via both GADD45B and TP53.** (**A**–**F**) In A375 cells, the CDKN1A expression after transfecting with miR-300 mimics and inhibitors, simultaneously transfecting with TP53 and GADD45B expression or knockdown vector. There was a blank control group and a negative control group. (-) represents gene interference and (+) represents gene expression. (**G**, **H**) In A375 cells, the cell cycle progress alteration after transfecting with miR-300 mimics and inhibitors, simultaneously transfecting with TP53 and GADD45B expression or knockdown vector. The proportion of cells in each group at different stages was calculated by the corresponding histogram. Blue represents the G1 phase, green represents the S phase, and red represents the G2 phase. * p-value < 0.05, ** p-value < 0.01, *** p-value < 0.001.

Since CDKN1A is a downstream target of TP53 and GADD45B. To confirm that miR-300 can regulate CDKN1A expression through TP53 and GADD45B, it was found that miR-300 indirectly changed the level of intracellular CDKN1A by posttranscriptional inhibit the expression of TP53 or GADD45B (Figure 7). The overexpression or interference vector of TP53 and GADD45B were transfected simultaneously in melanoma cells. It was found that TP53 regulated CDKN1A earlier than GADD45B and the regulation effect was more significant, presumably due to TP53’s transcription factor activity and higher affinity with downstream genes (Figure 7A–7F). As well known, CDKN1A is a classical cyclically dependent kinase inhibitor that effectively blocks cell cycle progression. Flow cytometry detection revealed that in melanoma cells, TP53 and GADD45B may mediate miR-300 regulating the expression of CDKN1A, and further promoting the cell cycle transition from the G1/S phase to the G2 phase (Figure 7G, 7H).

### MiR-300 regulates tumor growth through CDKN1A

**Figure 8:**
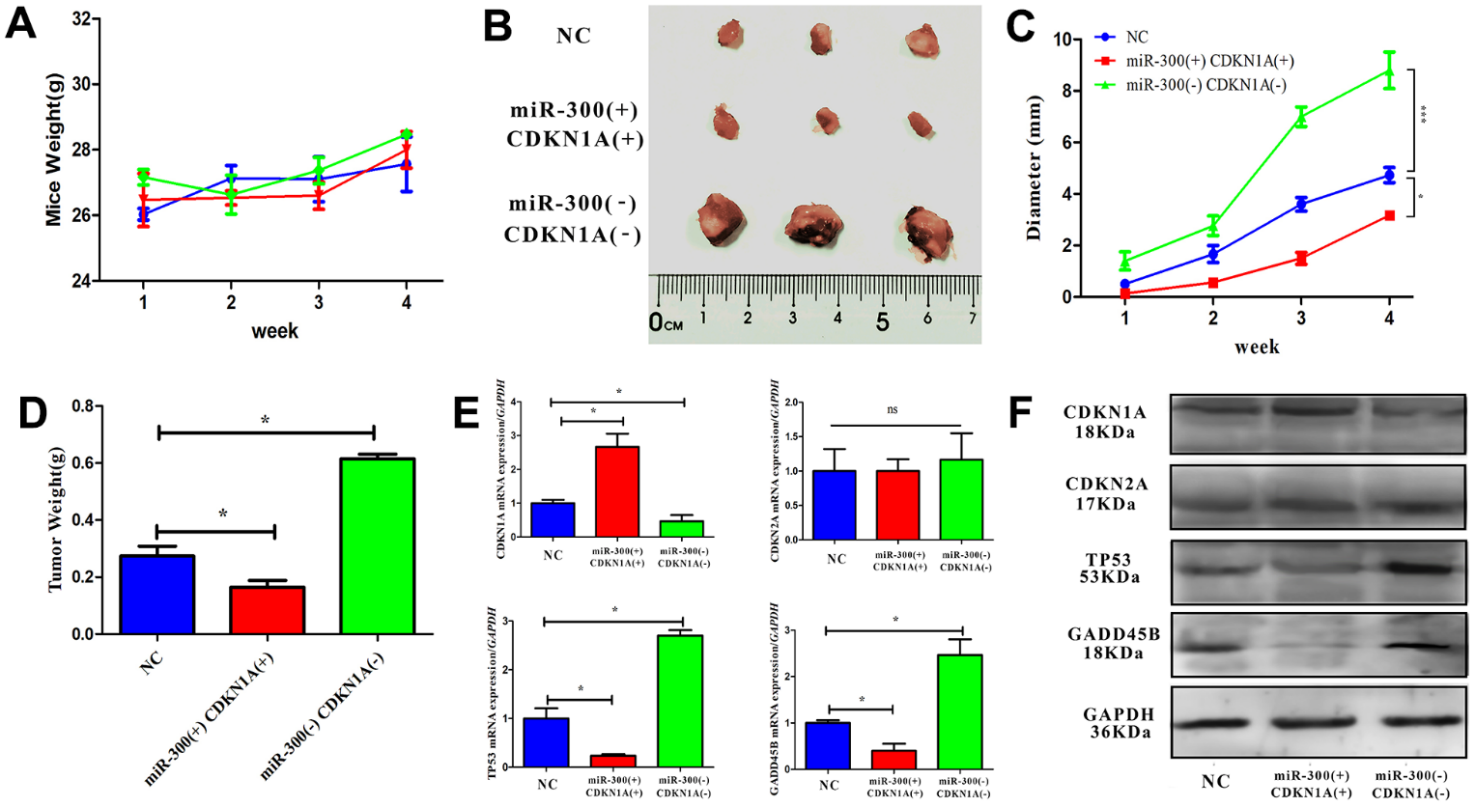
***In vivo*, hsa-miR-300 regulates tumor growth through CDKN1A.** (**A**) After the treated cells were inoculated, the weight of the mice was measured weekly. (**B**) Four weeks later, the mice were sacrificed to separate the tumor tissue, the diameter was measured and photos were taken. (**C**, **D**) After inoculation, tumor diameter and weight were measured weekly during tumor growth using vernier calipers and scale. (**E**, **F**) The expression of CDKN1A, CDKN2A, TP53 and GADD45B in tissue homogenate was detected. A total of 9 nude mice were included in the experiment and divided into control groups, miR-300 (-)/CDKN1A (-) and miR-300 (+)/CDKN1A (+) groups, where (-) represents gene interference and (+) represents gene expression. Each mouse was inoculated with 5 x 10^6^-1 x 10^7^ cells for axillary injection. * p-value < 0.05, ** p-value < 0.01, *** p-value < 0.001.

CDKN1A is a key factor in regulating the progress of the G1/S phase of the cell cycle. Abnormal expression of CDKN1A will directly affect the cell cycle. *In vitro* experimental results, miR-300 may indirectly inhibit CDKN1A expression through TP53 and GADD45B. To confirm the carcinogenic effect of miR-300 *in vivo*, a nude mouse model with heterograft was constructed. The weight and tumor diameter of the mice were measured weekly after tumor cell injection. During the experiment, the body weight of the mice did not fluctuate significantly and remained between 25 and 30 g (Figure 8A). After 4 weeks, the tumor tissue was taken out for diameter measurement and weight measurement (Figure 8B). Compared with normal control group, the tumors in miR-300(+)CDKN1A(+) were smaller (diameter=2.5 mm) while those in miR-300(-)CDKN1A(-) were larger (diameter=8.3 mm). The expression level of CDKN1A is up-regulated, which can effectively weaken the promoting effect of miR-300 on tumor growth (about 2 mm) (Figure 8C, 8D). The mRNA and protein levels of CDKN1A, CDKN2A TP53 and GADD45B in tumor tissues were detected after the tissue was broken. Compared with the control group, the expressions of miR-300 and CDKN1A were up-regulated simultaneously (p-value<0.05), and CDKN2A was not significantly changed (p-value>0.05). TP35 and GADD45B were down-regulated by miR-300 (p-value<0.05) and vice versa (Figure 8E, 8F).

## DISCUSSION

The activation state of the cell cycle pathway is one of the key factors affecting tumor growth and malignancy. It has been preliminarily confirmed that miR-300-GADD45B can promote the melanoma cell cycle [34]. However, the specific mechanism was still unclear. In primary and metastatic melanoma with different degrees of malignancy, molecular differences were screened and functional analysis was performed. It was found that in metastatic melanoma, up-regulated genes were involved in cancer-related functions (pathways in cancer and microRNAs in cancer), while down-regulated genes were involved in maintaining skin development and homeostasis (keratinization, keratinocyte differentiation, epidermis development and skin development). In Li et al. study, by analyzing the DEGs between metastatic and nonmetastatic melanoma and constructing the protein-protein interaction (PPI) network, it was found that as proof of the differential expression of metastasis-associated genes, 11 keratinocyte differentiation-involved genes, including LOR, EVPL, SPRR1A, FLG, SPRR1B, SPRR2B, TGM1, DSP, CSTA, CDSN, and IVL, were down-regulated in metastatic melanoma in comparison with primary melanoma based on the data from TCGA [39]. However, the crosstalk between melanoma cells and keratinocytes was bidirectional. In Kodet et al. study, comparative morphometrical and immunohistochemical analysis of epidermis surrounding nodular melanoma (n=100) was performed. The epidermis surrounding nodular melanoma exhibits hyperplastic features in 90% of cases. This hyperplastic region exhibits aberrant suprabasal expression of keratin 14 accompanied by loss of keratin 10. It was concluded that the melanoma cells are able to influence locally the differentiation pattern of keratinocytes [40].

Furthermore, 4 melanoma pathway genes (CDKN1A, CDKN2A, CXCR4 and RAD51) were also expressed differently in normal tissues compared with melanoma. Only CDKN1A and CDKN2A were directly involved in TP53-dependent cell cycle regulation pathways [41–43]. Interestingly, in our previous study, it was confirmed that in melanoma, the miR-300 could regulate the expression of GADD45B which would also mediate cell cycle pathway [44, 45]. Therefore, it was hypothesized that CDKN1A, CDKN2A and TP53 might interact with miR-300 and GADD45B. By constructing the transcription factor-miRNA-target gene network, it was found that TP53 was not only the upstream transcription factor of miR-300 but also negatively regulated by it. In this study, due to the focus of our research was cell cycle, there is not much attention paid to the other 2 DEGs, which does not mean that they are not important. On the contrary, many studies have confirmed their role in the development of melanoma. For example, the chemokine receptor CXCR4 is active in melanoma metastasis. CXCR4 expression is driven through a highly conserved intronic enhancer element by the transcription factors PAX3 and FOXD3. Inhibition of these transcription factors slows melanoma cell growth, migration, and motility, as well as reduces CXCR4 expression [46]. RAD51 is an essential factor of the homologous recombination DNA repair pathway and therefore plays an important role in maintaining genomic stability. In Makino et al. study, RAD51 inhibitors could lead to DNA damage, G2/M arrest and apoptosis in melanoma [47].

In addition, miR-300 can down-regulate the expression of CDKN1A in melanoma cells by regulating TP53 and GADD45B, thus promoting the transition of the cell cycle from the G1/S phase to the G2 phase and accelerating tumor growth. In addition, although GADD45A, which belongs to the same GADD45 family, has been confirmed to be regulated by TP53 [48, 49], GADD45B in melanoma is not regulated by TP53 in this study. Meanwhile, it was confirmed that in melanoma, CDKN1A and CDKN2A could not affect the miR-300 transcription and maturation process. In conclusion, there is a signal axis regulating the cell cycle in melanoma. The miR-300 and CDKN2A are upstream of this signal axis affecting the expression of TP53 and GADD45B, while CDKN1A is downstream mediating the promoting effect of miR-300 on the cell cycle.

In present study, the results showed that the expression level of CDKN1A was down-regulated with tumorigenesis. At the same time, when comparing the transcriptomes of primary and metastatic melanoma, CDKN1A was also down-regulated in metastatic melanoma which has higher malignancies (Figure 2). All these suggest that CDKN1A can be used as a tumor suppressor gene in melanoma. However, when we plotted a survival curve based on the clinical information of melanoma patients in the TCGA database. It was found that patients had a worse prognosis when CDKN1A was at a higher level. It seems to indicate that CDKN1A is an oncogene, which is contrary to the conclusion of this study. In response to this phenomenon, we have reviewed a lot of literature. Almost all studies about CDKN1A and melanoma suggest that the up-regulation of CDKN1A expression can inhibit the growth of melanoma cells, accelerate aging, and improve the sensitivity of therapeutic drugs [50, 51]. Of course, there are also some studies to the contrary. In Shi et al. study, it was found lycorine hydrochloride (LH), an active ingredient sourced from the medicinal herb lycoris radiate, reduced p21Cip1/WAF1 protein by accelerating its ubiquitination and suppresses the proliferation and metastasis of melanoma cells [52].

## Supplementary Material

Supplementary Figures
