# Correction for: Has-miR-300—GADD45B promotes melanoma growth via cell cycle

**DOI:** 10.18632/aging.205515

**Published:** 2024-01-14

**Authors:** Long Chen, Chenglong Fang, Xiaoxue Yuan, Mengqi Liu, Ping Wu, Li Zhong, Zhiyong Chen

**Affiliations:** 1 Department of Burn Plastic and Cosmetology, Affiliated Fuling Hospital, Chongqing University, Chongqing 408099, China; 2 College of Bioengineering, Chongqing University, Chongqing 400000, China; 3 Department of Immunology, School of Basic Medical Sciences, Chengdu Medical College, Chengdu, Sichuan 610500, China; 4 Non-Coding RNA and Drug Discovery Key Laboratory of Sichuan Province, Chengdu Medical College, Chengdu Sichuan 610500, China; 5 Department of Rehabilitation, Linyi People’s Hospital, Linyi, Shandong 276000, China

**Keywords:** miR-300, GADD45B, CDKN1A, cell cycle, melanoma

**This article has been corrected:** The authors corrected the listed affiliations by combining affiliations 1 and 2 into affiliation 1 after those two units officially established a partnership and merged into a single unit. They also acknowledged Dr. Li Zhong as a co-corresponding author. The correct authors list with the corresponding affiliations is presented below.

Long Chen^1,2,3,*^, Chenglong Fang^4,*^, Xiaoxue Yuan^1,*^, Mengqi Liu^1^, Ping Wu^1^, Li Zhong^1,#^,  Zhiyong Chen^1,#^

^1^College of Bioengineering and Department of Burn Plastic and Cosmetology, Affiliated Fuling Hospital, Chongqing University, Chongqing 408099, China

^2^Department of Immunology, School of Basic Medical Sciences, Chengdu Medical College, Chengdu 610500, Sichuan, China

^3^Non-Coding RNA and Drug Discovery Key Laboratory of Sichuan Province, Chengdu Medical College, Chengdu 610500, Sichuan, China

^4^Department of Rehabilitation, LinYi People’s Hospital, Linyi 276000, Shandong, China

*Equal contribution

^#^Co-corresponding authors

**Correspondence to:** Li Zhong, Zhiyong Chen; **email:** jlzhong@cqu.edu.cn, czy1002008@cqu.edu.cn

