# Supplementary material for: Has-miR-300—GADD45B promotes melanoma growth via cell cycle
Source: Aging (Albany NY). 2023 Dec 7;15(23):13920–43. doi: 10.18632/aging.205276 (PMC10756120; doi:10.18632/aging.205276)
Supplement: Supplementary Figures [file aging-15-205276-s001.pdf]

SUPPLEMENTARY FIGURES

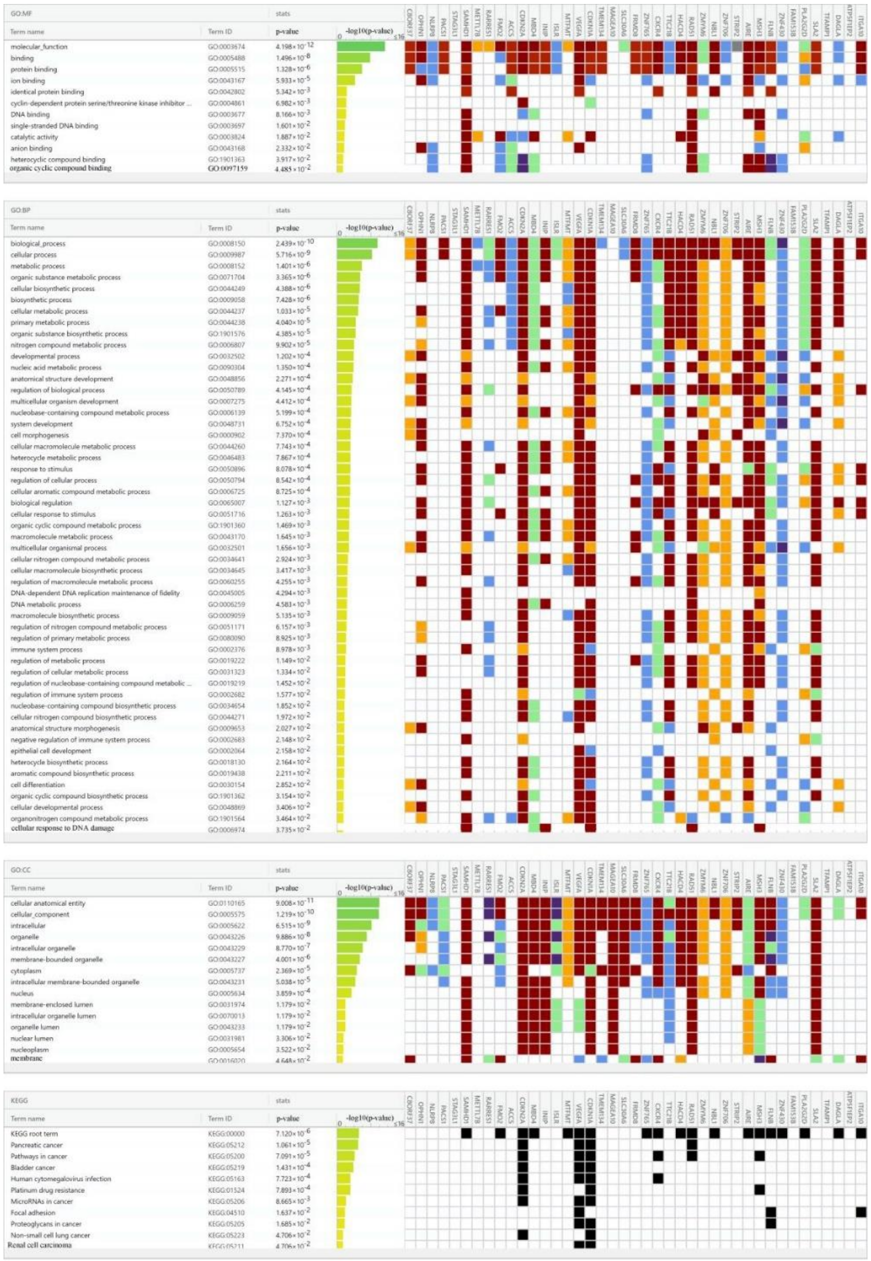

Supplementary Figure 1. The enrichment analysis of 42 up-regulated genes in metastatic melanoma. In this chart, the GO (MF, BP and CC) and KEGG items that are significantly enriched were listed from the top to bottom. The information contained is mainly: term name, term ID, adjusted p-value, negative log10 of adjusted p-value and correlated genes. In the negative log10 of adjusted p-value column, the color (blue>green>yellow) and length of the bar represented the size of the p-value, as well as significance. In the rear grid, colored squares indicated that the single gene was involved in this GO function or pathway. The different color represents different verification methods: red squares represent inferred from experiment [IDA, IPI, IMP, IGI, IEP], direct assay [IDA], mutant phenotype [IMP], genetic interaction [IGI] and physical interaction [IPI]; blue squares represent inferred from high throughput experiment; green squares represent Traceable author [TAS], Non-traceable author [NAS], Inferred by curator [IC]; yellow squares represent expression pattern [IEP], sequence or structural similarity [ISS], genomic context [IGC], sequence model [ISM], sequence alignment [ISA], sequence orthology [ISO], biological aspect of ancestor [IBA] and rapid divergence [IRD]; azure squares represent reviewed computational analysis [RCA], electronic annotation [IEA]. In addition, the blank squares indicated that the gene is not correlated to the enriched function or pathway.

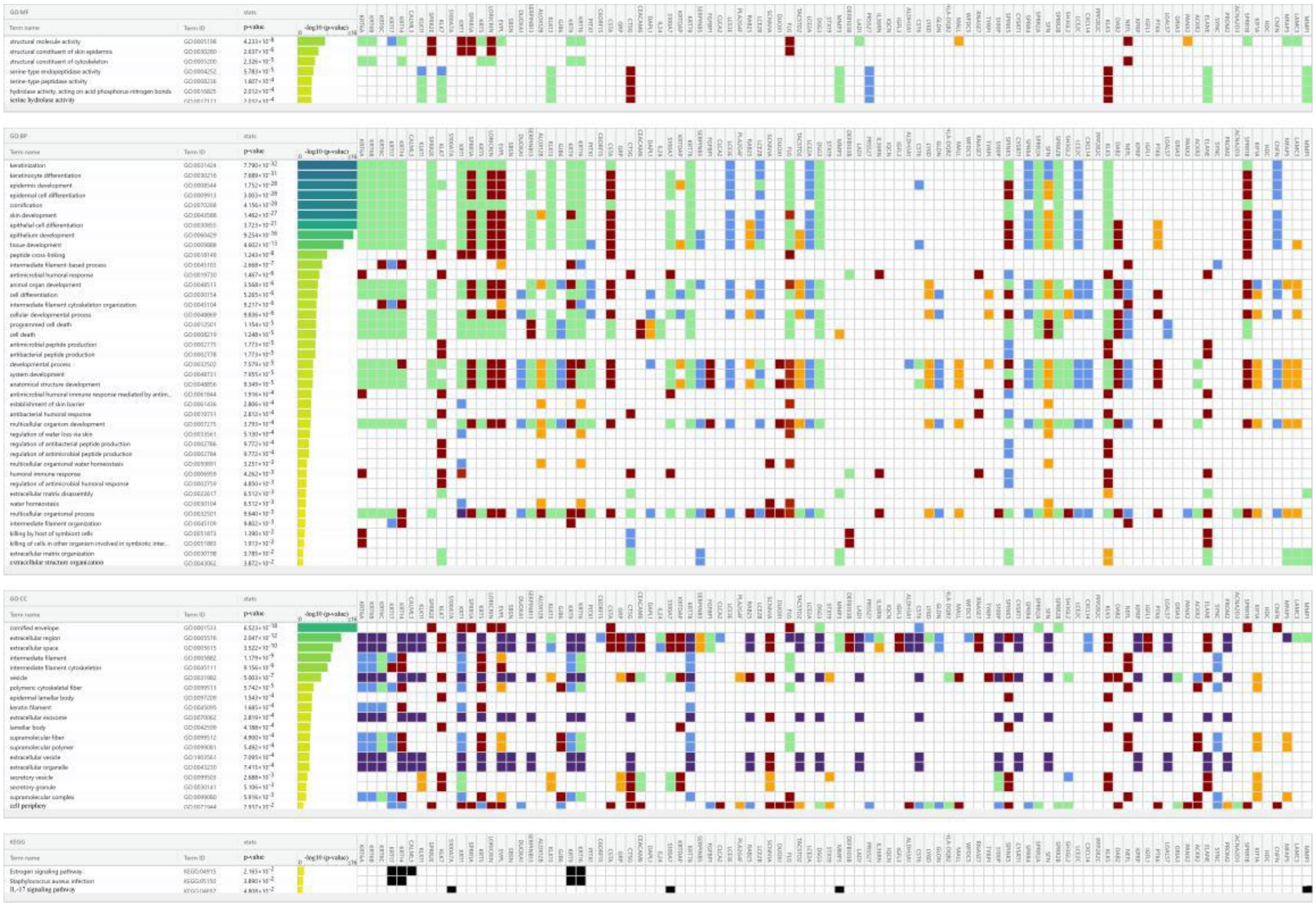

Supplementary Figure 2. The enrichment analysis of 97 down-regulated genes in metastatic melanoma. In this chart, the GO (MF, BP and CC) and KEGG items that are significantly enriched were listed from the top to bottom. The information contained is mainly: term name, term ID, adjusted p-value, negative log10 of adjusted p-value and correlated genes. In the negative log10 of adjusted p-value column, the color (blue>green>yellow) and length of the bar represented the size of the p-value, as well as significance. In the rear grid, colored squares indicated that the single gene was involved in this GO function or pathway. The different color represents different verification methods: red squares represent inferred from experiment [IDA, IPI, IMP, IGI, IEP], direct assay [IDA], mutant phenotype [IMP], genetic interaction [IGI] and physical interaction [IPI]; blue squares represent inferred from high throughput experiment; green squares represent Traceable author [TAS], Non-traceable author [NAS], Inferred by curator [IC]; yellow squares represent expression pattern [IEP], sequence or structural similarity [ISS], genomic context [IGC], sequence model [ISM], sequence alignment [ISA], sequence orthology [ISO], biological aspect of ancestor [IBA] and rapid divergence [IRD]; azure squares represent reviewed computational analysis [RCA], electronic annotation [IEA]. In addition, the blank squares indicated that the gene is not correlated to the enriched function or pathway.

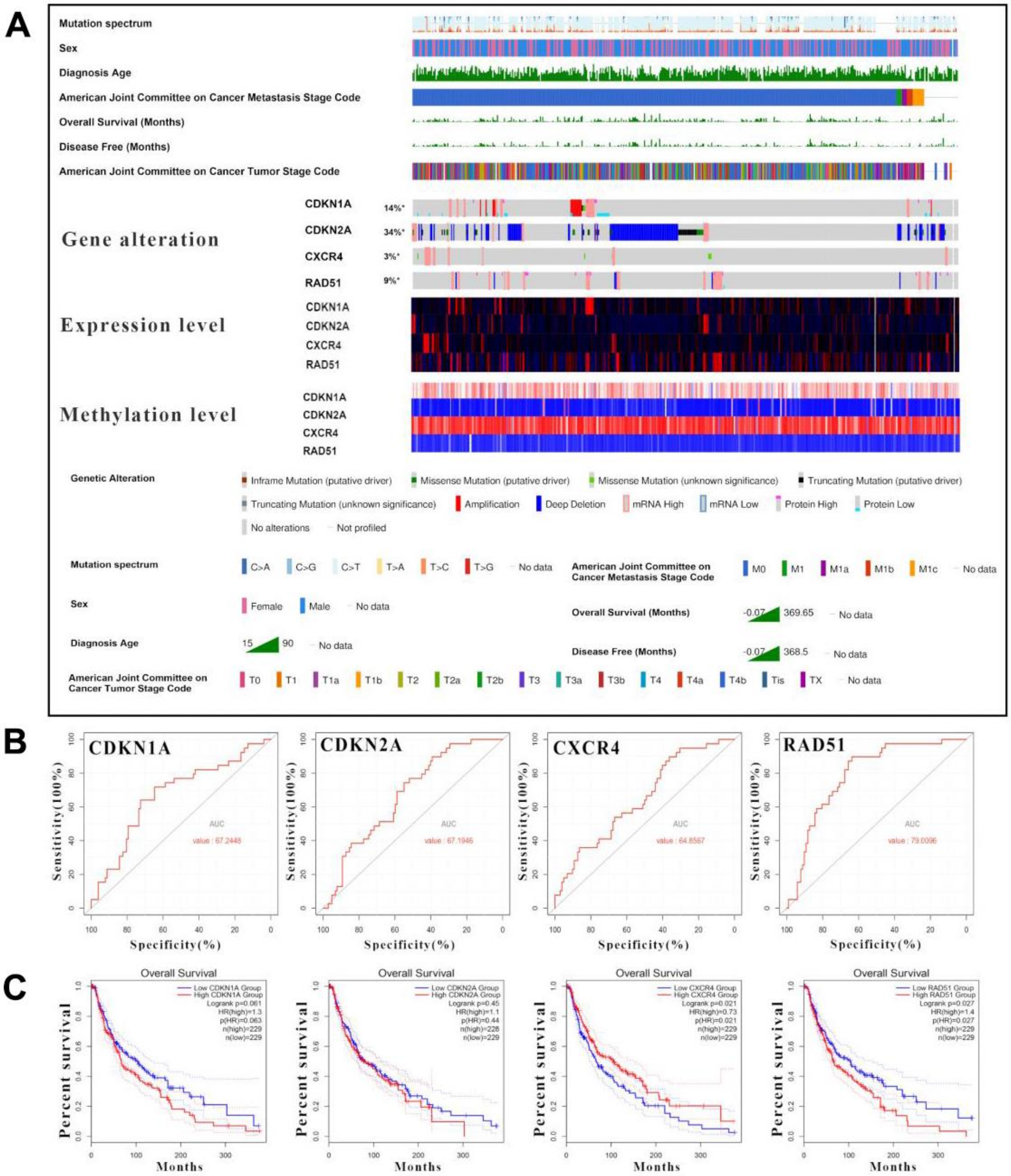

Supplementary Figure 3. The clinical correlation of CDKN1A, CDKN2A, CXCR4 and RAD51. (**A**) The molecular distortion panorama of 4 key genes in 472 melanoma samples with clinical data from TCGA. From top to bottom panels indicate: mutation spectrum, sex, diagnosis age, American Joint Committee on Cancer metastasis stage code, overall survival, disease free, American Joint Committee on Cancer tumor stage code, the heatmap of gene alteration, expression level and methylation of CDKN1A, CDKN2A, CXCR4 and RAD51, GADD45B. The key to the color-coding is at the bottom. (**B**) The ROC curves of 4 key genes in melanoma patients. (**C**) The survival curves of 4 key genes between 2 groups with high or low expression levels, red lines represent high expression level, blue lines represent low expression level. The logrank p-value, HR (high) and p-value (HR) were listed.

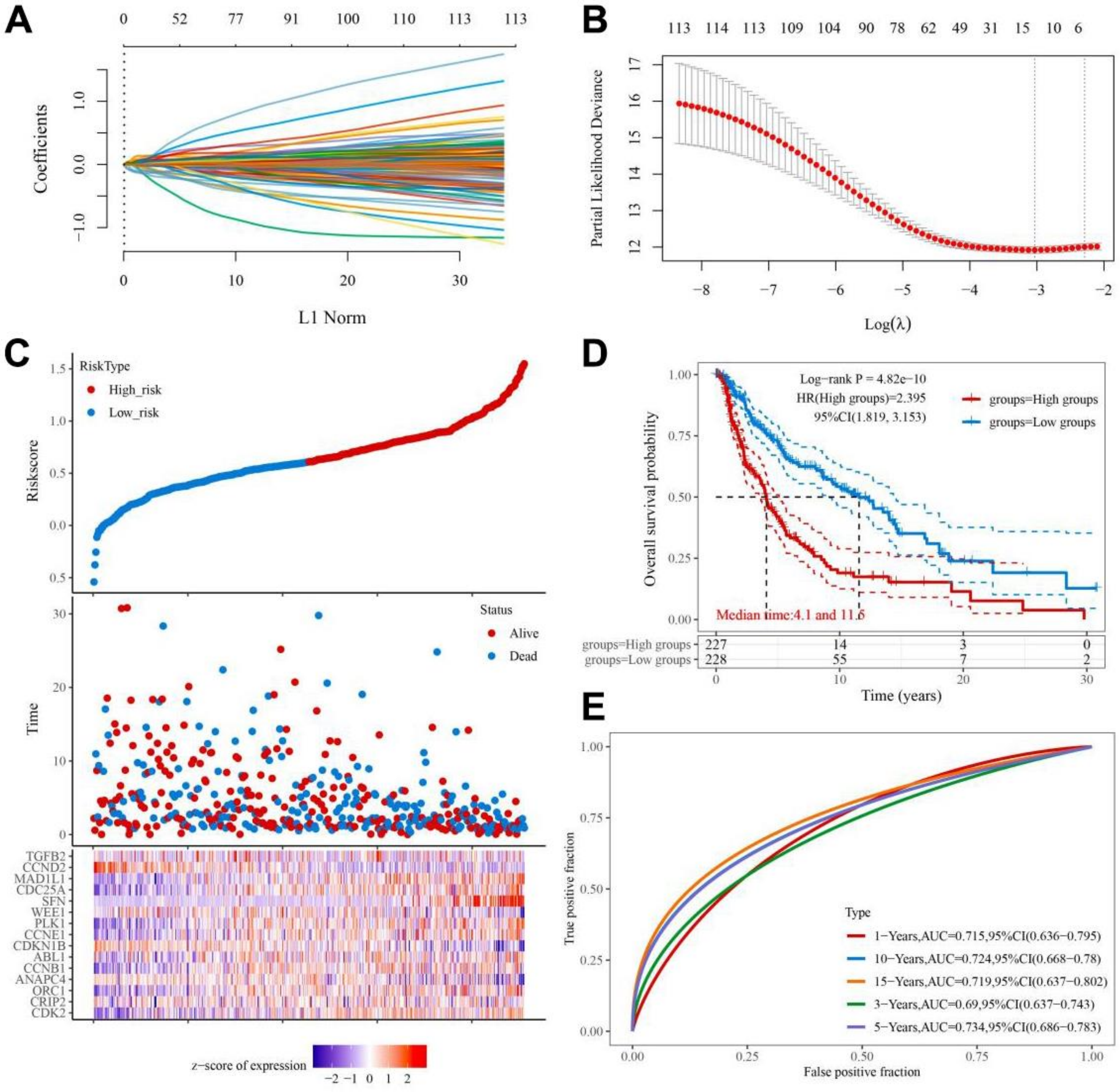

Supplementary Figure 4. The prognostic analysis of GADD45B molecular network (13-gene signature). (**A**) LASSO coefficients profiles of genes in GADD45B network. (**B**) LASSO regression with tenfold cross-validation obtained 13 prognostic genes using minimum lambda value. (**C**) From top to bottom: The curve of risk score; the survival status of the patients. More dead patients corresponding to the higher risk score; the heatmap of the expression profiles of the 13 prognostic genes in low- and high-risk group. (**D**) Kaplan–Meier survival analysis of the 13-gene signature. (**E**) Time-dependent ROC analysis of the 13-gene signature. ROC receiver operating characteristic.

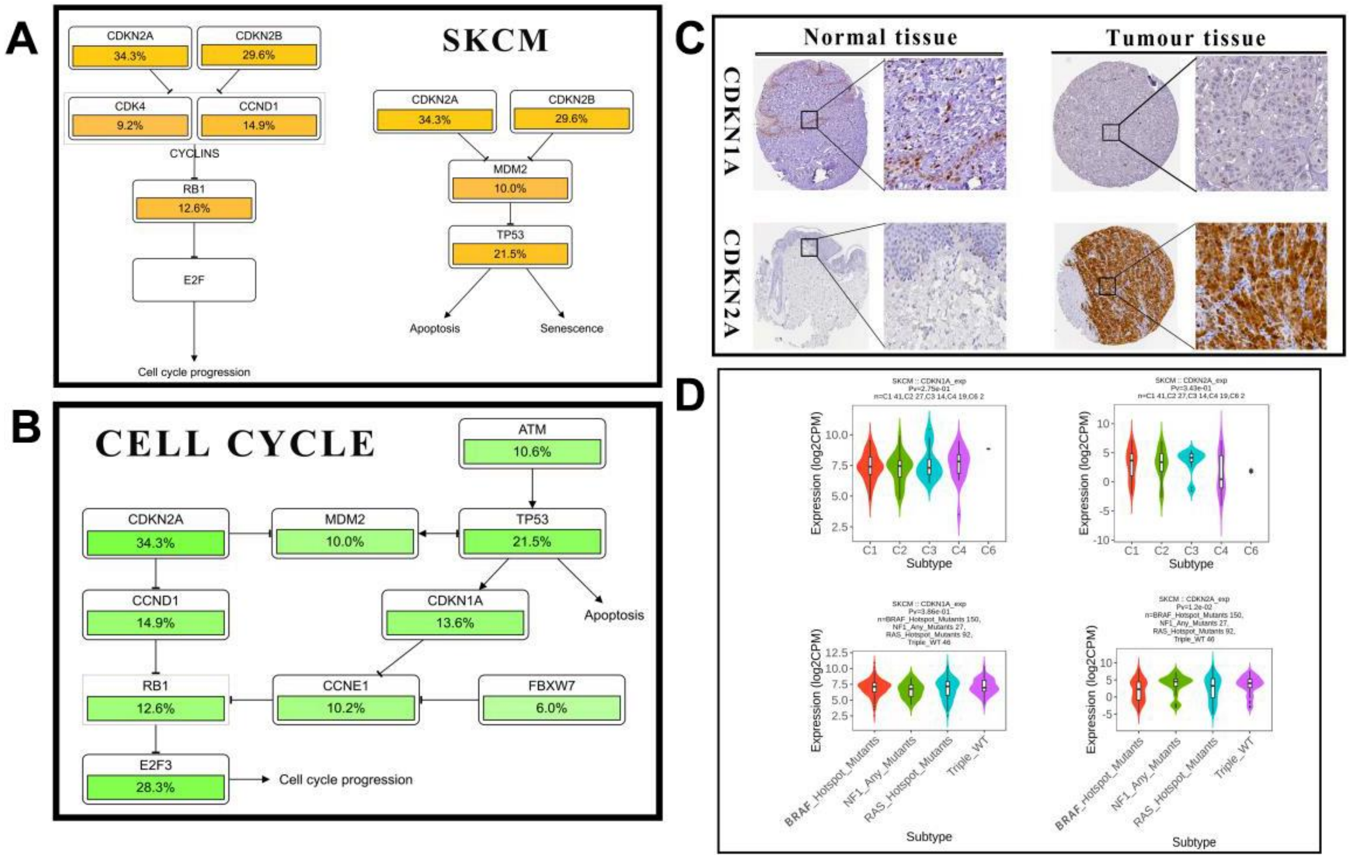

Supplementary Figure 5. The role of CDKN1A and CDKN2A in melanoma. (**A**, **B**) The schematic diagrams show the location and downstream genes of CDKN1A and CDKN2A in the melanoma signaling pathway and cell cycle signaling pathway, respectively. The aberration frequency of each gene in melanoma patients was also marked. (**C**) The protein level of CDKN1A and CDKN2A in normal and melanoma tissues. The pictures of immunohistochemistry results with different magnifications. Brown represents the target protein. (**D**) The violin plots represent the expression of CDKN1A and CDKN2A in various subtypes, such as BRAF hotspot mutants, NF1 any mutants and triple WT.
